# Gene Expression Analysis of Children with Acute Hematogenous Osteomyelitis Caused by Methicillin-Resistant *Staphylococcus aureus*: Correlation with Clinical Severity of Illness

**DOI:** 10.1371/journal.pone.0103523

**Published:** 2014-07-30

**Authors:** Claudia Gaviria-Agudelo, Kristen Carter, Naureen Tareen, Virginia Pascual, Lawson A. Copley

**Affiliations:** 1 Department of Pediatrics Infectious Disease, University of Texas Southwestern Medical Center, Dallas, Texas, United States of America; 2 University of Texas Southwestern Medical Center, Dallas, Texas, United States of America; 3 Children’s Medical Center, Dallas, Texas, United States of America; 4 Baylor Institute for Immunology Research, Dallas, Texas, United States of America; 5 Orthopaedic Surgery, University of Texas Southwestern Medical Center, Dallas, Texas, United States of America; 6 Texas Scottish Rite Hospital, Dallas, Texas, United States of America; Kent State University, United States of America

## Abstract

Children with acute hematogenous osteomyelitis (AHO) demonstrate a broad spectrum of clinical manifestations, ranging from mild to severe. Several advances have been achieved in the study of host immune response to acute invasive *Staphylococcus aureus* infections through gene expression analysis. However, previous research has neither attempted to evaluate the response of children with AHO specific to Methicillin-resistant *Staphylococcus aureus* (MRSA) nor to correlate gene expression with clinical phenotype. Study objective was to correlate gene expression of children with AHO due to MRSA with clinical severity of illness. Whole blood samples were obtained in Tempus tubes from 12 children with osteomyelitis once cultures obtained directly from the site of infection confirmed to be positive for MRSA. Using an Illumina platform and a systems-wide modular analysis, microarray findings from ten of these children were compared to that of nine healthy (age, ethnicity and gender) matched controls and correlated with clinical severity of illness. Children with AHO from MRSA demonstrated over-expression of innate immunity with respect to neutrophil activity, coagulation, inflammatory response, and erythrocyte development. Concurrently, these children demonstrated under-expression of adaptive immunity with respect to lymphocyte activation and activity of T-cell, cytotoxic or NK cell, and B-cell lines. Three over-expressed genes, P2RX1, SORT1, and RETN, and two under-expressed genes, LOC641788 and STAT 4, were significantly correlated with severity of illness. STAT 4 showed the strongest correlation (R2 = –0.83). STAT4 downregulation could potentially explain under-expression of genes related to adaptive immunity in this cohort of patients with AHO. This study identified specific genes which correspond to disease severity during the early hospitalization of children with AHO from MRSA. Pattern recognition of this combination of genes could help to identify in the future severe clinical phenotypes before the disease is fully manifest and direct appropriate attention and resources to those children.

## Introduction

Children with acute hematogenous osteomyelitis (AHO) demonstrate a broad range of clinical manifestations. [1]–[6] Those with mild illness respond quickly to antibiotic therapy, which can be accomplished at home following brief hospitalization. [1] In contrast, other children demonstrate severe illness that necessitates intensive care, multiple surgical interventions, and prolonged hospitalization. [2], [3], [7] This variability occurs in otherwise healthy, immune competent children, even if the causative organism is similar between cases. The underlying genetic mechanisms which may explain the diversity of clinical presentation are incompletely understood.

Gene-expression analysis has been used to study human transcriptional response in cancer, influenza, systemic lupus erythematosus (SLE) and infectious diseases. [8]–[11] Microarray analysis has led to a greater understanding of the diagnostic signature, which may be formed by expressed RNA of individuals who have a specific disease when compared to that of either healthy control subjects or children with other diseases. [8]–[14] Previous research has identified transcriptional signatures associated with *Staphylococcus aureus* invasive infection using peripheral blood mononuclear cells (PBMCs). [12], [13] One group found that children with *S. aureus* infections demonstrate a distinctive gene expression profile reflecting increased neutrophil activity that differentiates them from children with *Escherichia coli* infections. [12] Subsequently, these investigators applied a module-level analysis framework and confirmed that invasive *S. aureus* infections are associated with over-expression of innate immunity, characterized by increased transcription of genes related to neutrophil and monocyte activity, and under-expression of adaptive immunity, characterized by decreased transcription of genes related to T-cell and natural killer cell activity [13], [14].

Previous studies in this area have been conducted in diverse populations of children with a variety of infection types (skin and soft tissue, invasive, and disseminated); organ systems involved (osteoarticular, pulmonary, cardiac and lymphatic); and causative organisms (Methicillin-sensitive and Methicillin-resistant *S. aureus* (MSSA and MRSA)). [12]–[14] No previous study has focused exclusively on children with AHO caused by MRSA. The purpose of this study was to describe the gene expression pattern specific to these children and to assess the correlation of host gene quantitative over- and under-expression with clinical severity of illness in this homogenous population.

## Methods

### Ethics Statement

This study was conducted according to the principles expressed in the Declaration of Helsinki. The study was approved by the Institutional Review Board of the University of Texas Southwestern Medical Center Dallas and Children’s Medical Center of Dallas (IRB #STU 062011-009). Written informed consent was obtained from legal guardians of the patient and informed assent was obtained from patients 10 years of age and older prior to any study-related procedure.

### Patient Population

Previously healthy children who were admitted to the hospital with AHO due to MRSA were consecutively enrolled and prospectively studied between 2010 and 2011. AHO was defined as an infection involving bone diagnosed within 2 weeks of the onset of symptoms. The infection was acquired by hematogenous dissemination as opposed to direct inoculation of the bone due to trauma or surgery. The diagnosis was established by the combination of magnetic resonance imaging (MRI) findings, elevation of inflammatory markers (C-reactive protein and erythrocyte sedimentation rate), and the results of blood and bone tissue cultures. Following culture confirmation of MRSA, whole blood samples were collected from the children in Tempus tubes and stored frozen (–80°C) at the Texas Scottish Rite Hospital genetics laboratory. Children were excluded from the investigation if they had any underlying medical disorder which may lead to immune compromise such as: congenital immune deficiency, leukemia, organ transplant, or treatment with chemotherapy or immunomodulatory agents. Also excluded were children with infection due to any bacterial organism other than MRSA.

### Demographic and Clinical Data

The following clinical and laboratory data were gathered: age; gender; ethnicity; infection site; number of surgeries; total length of stay (including pediatric intensive care unit stay and readmission days); and severity of illness scoring parameters. According to our previously published protocol [15], a severity of illness score was calculated for each child based on the following clinical and laboratory data: C-reactive protein (CRP) values at admission, 48 hours, and 96 hours; febrile days on antibiotics; admission respiratory rate; and evidence of disseminated disease (multi-focal involvement, deep venous thrombosis, pulmonary involvement, meningitis, or endocarditis).

### RNA preparation and Microarray Hybridization

Whole blood mRNA samples were thawed and studied at the Baylor Institute for Immunology Research. Comparison was made with age, gender and ethnicity matched healthy control subjects within the Baylor database. Microarray analysis on Illumina v6 chips was performed on whole blood RNA samples.

#### Illumina

Samples were processed and data acquired by Illumina (San Diego, CA). Targets were prepared using the Illumina RNA amplification kit (Ambion, Austin, TX). cRNA was obtained from 200 ng of total RNA and after in vitro transcription underwent amplification and labeling steps. Amplified biotin-labeled cRNA was hybridized to the Illumina Sentrix Hu6 BeadChips (Ambion, Austin, TX).

#### Illumina BeadChips

The Sentrix Hu6 BeadChips consist of 50 mer oligonucleotide probes attached to 3–µm beads within microwells on the surface of the glass slide representing 48,687 probes. Slides were scanned on Illumina BeadStation 500 and Illumina Beadstudio software was used to assess fluorescent hybridization signals.

### Microarray Data Analysis

Microarray Suite, version 5.0 (MAS 5.0; Affymetrix) software was used to assess fluorescent hybridization signals, to normalize signals, and to evaluate signal detection calls. Raw signal intensity values for each probe set were analyzed by algorithms in MAS 5.0. Normalization of signal values per chip was achieved using the MAS 5.0 global method of scaling to the target intensity value of 500 per gene chip. GeneSpring, version 7.1 (Agilent) was used to perform statistical analysis, hierarchical clustering, and classification of samples.

### Modular Framework

An analytical framework of 62 transcriptional modules was assembled from study data and the respective healthy control group as a reference. These modules group genes with shared expression pattern and similar biological functions.

### Statistical Analysis and Correlation with Severity of Illness

Transcribed genes were sorted by hierarchical and conditional clustering, normalized to the healthy controls. The genes identified by hybridization were then evaluated with the Welch’s t-test using a p-value of 0.05 and multiple testing correction with the Benjamini and Hochberg False Discovery Rate to identify all transcribed genes which significantly differentiated children with MRSA AHO from children of the healthy control group. The quantitative over- and under- expression of the statistically significant genes was then correlated against the clinical severity of illness scores using four statistical correlation methods (Pearson, Spearman, Pearson Log Gene and Pearson Log Log).

## Results

**Figure 1:**
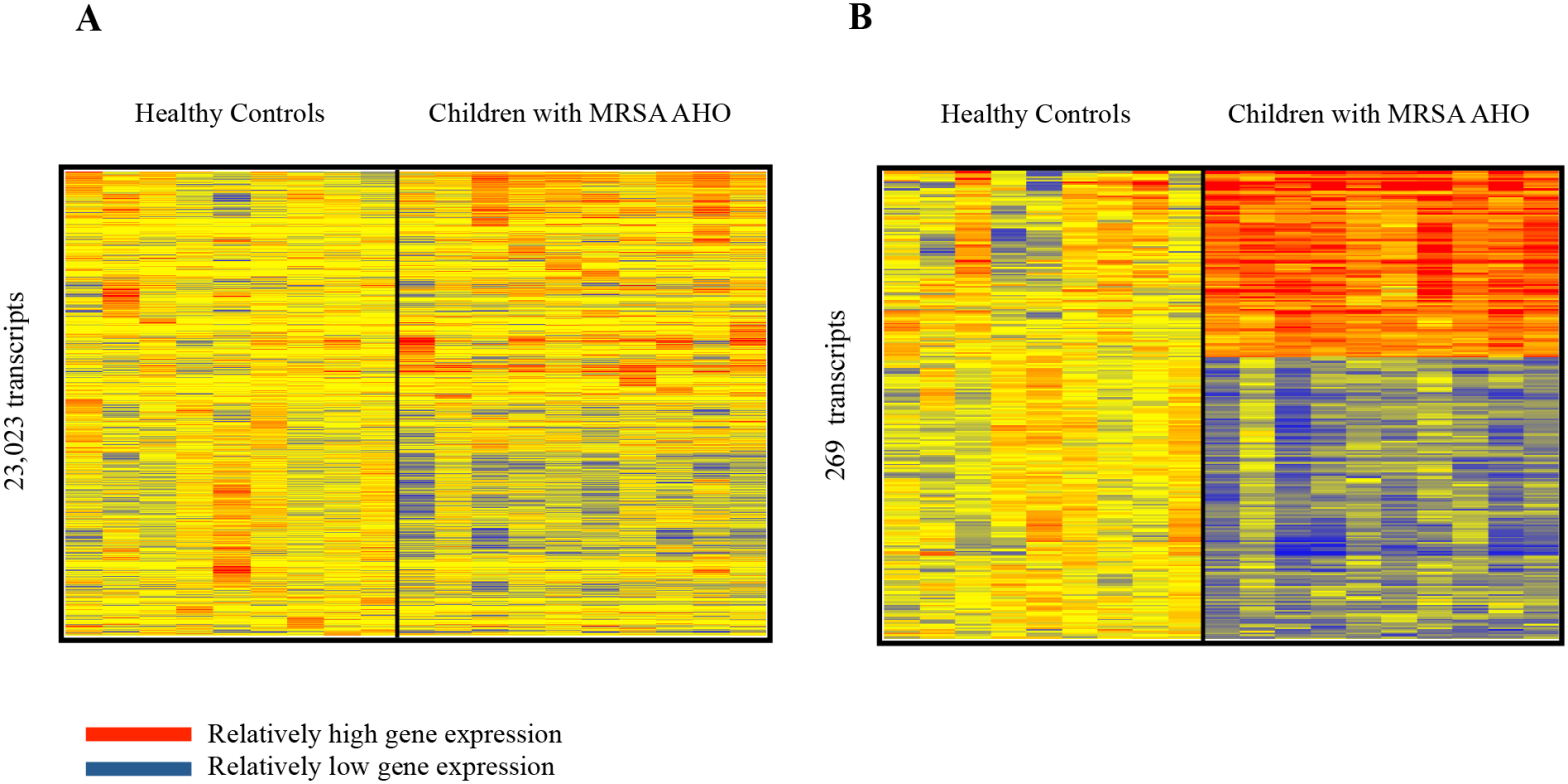
Gene Expression Biosignature in Whole Blood From Healthy Controls and Patients With MRSA AHO. Statistical tests were used to compare the two study groups (Welch’s t-test, p<0.05 with Benjamini-Hochberg’s method). **A)** Analysis of the healthy control group with 9 subjects and the MRSA AHO group with 10 patients yielded 23,023 genes expressed at statistically different levels. Hierarchical clustering, used to organize those genes to reveal differential expression, supports that patients with MRSA AHO have unique signatures that classify them as different from controls. **B)** Further statistical analysis between the healthy controls and the MRSA AHO group yielded 269 genes expressed at different levels.

**Figure 2:**
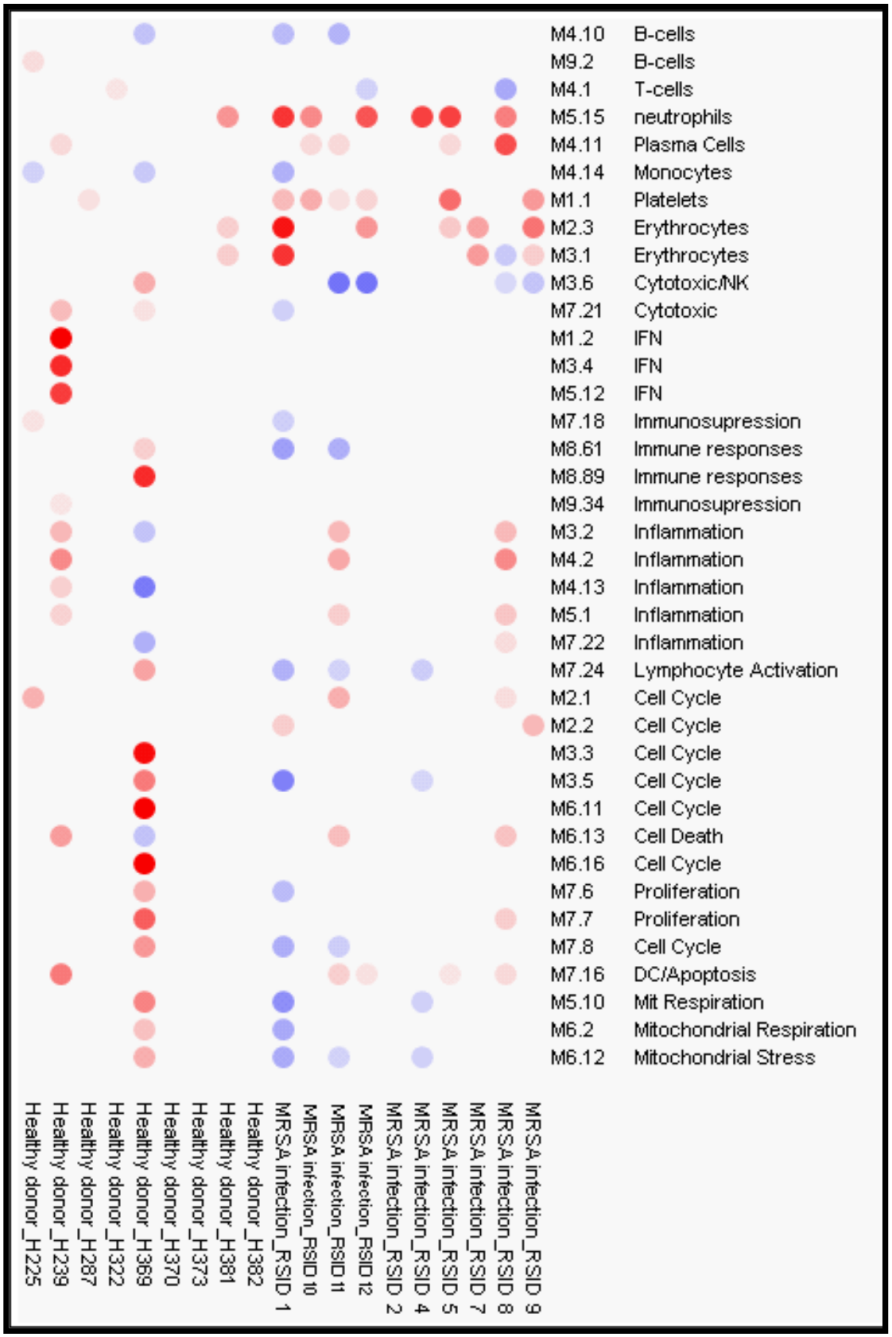
Module Analysis of Gene Expression Profile in Whole Blood From Patients with MRSA AHO. Gene expression in one of the subjects within the healthy control group revealed an unusual array of cellular expression. This subject was excluded and microarray statistical analysis was repeated without his information. Gene expression levels were compared between healthy controls and patients with MRSA AHO on a module by module analysis. Each column represents a subject and each row a cell type. Colored spots represent the percentage of significant over-expressed (red) or under expressed (blue) transcripts (Mann-Whitney p<0.05). The intensity of the color refers to the number of transcripts within each of the pre-selected modules not the actual fold change that is up or down regulated compared to healthy controls. Modular analysis identified that patients had upregulation of innate immunity and mild downregulation of adaptive immunity.

**Figure 3:**
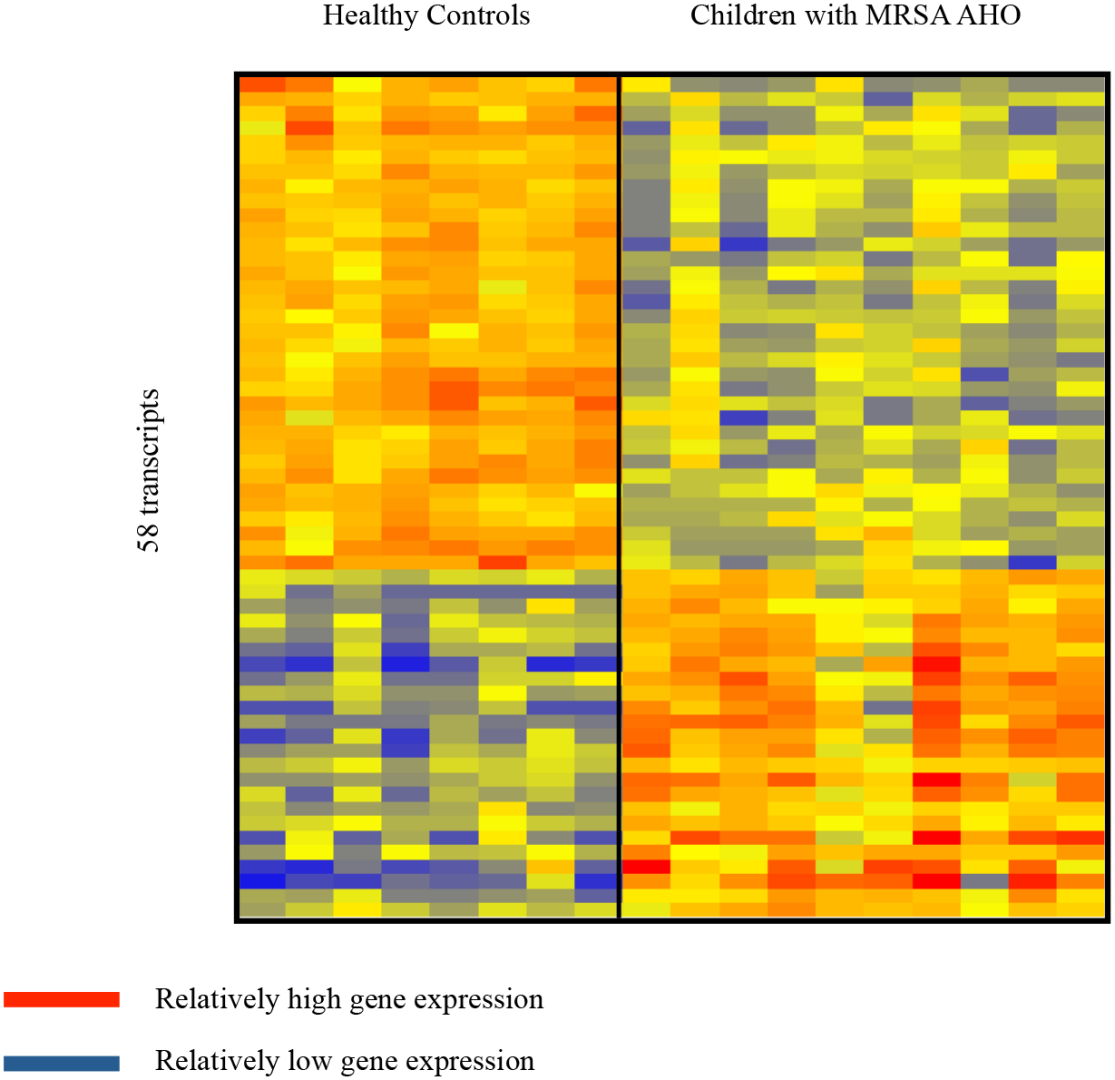
Gene Expression Biosignature in Whole Blood From Healthy Controls and Patients With MRSA AHO. Complete statistical analysis (Welch t-Test with Benjamini - Hochberg false discovery rate, p<0.05) between the two groups (healthy control group with 9 subjects and MRSA AHO group with 10 subjects) identified 58 genes (24 over-expressed and 34 under-expressed) which significantly differentiated children with MRSA AHO from the healthy controls.

**Figure 4:**
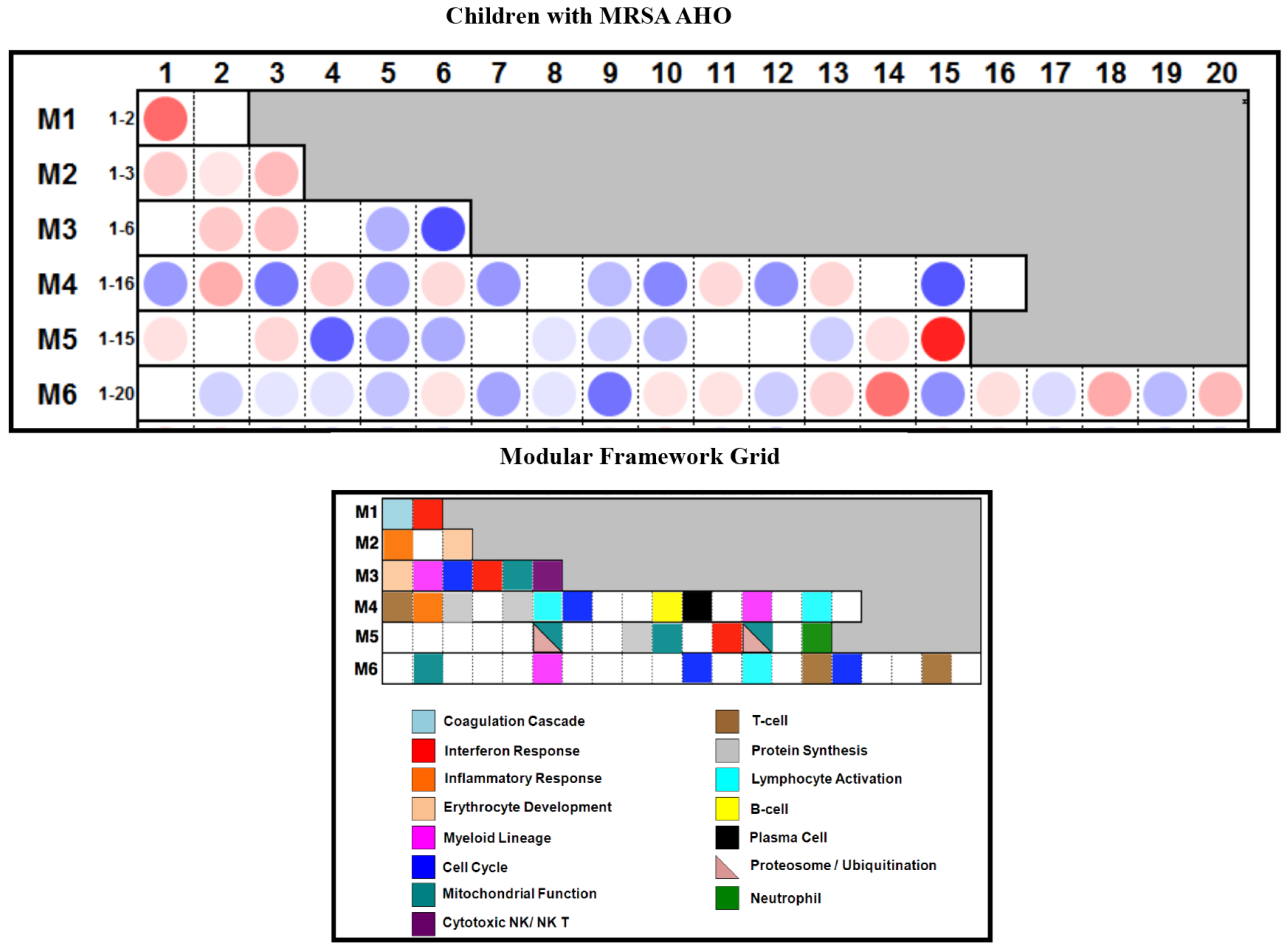
Module Analysis of Gene List Applied To an Independent Set of Patients Revealed The Same Modular Map. Average modular transcriptional fingerprint of patients with MRSA AHO showed that patients had upregulation of innate immunity including platelet, neutrophil and erythroid-related genes. Analysis also showed that patients had mild downregulation of adaptive immunity including B- cell, T-cell and NK cell-related genes. Interferon signature was not noted in this analysis.

12 children with MRSA AHO were enrolled during the study. The clinical data of these children are shown in Table 1. Matched control samples were unavailable for 2 children due to their young age (<2 years) so their samples were excluded from subsequent analysis. Fluorescent hybridization resulted in the identification of 23,023 transcripts within the microarray of the remaining 10 children (Figure 1A). Welch’s t-test with multiple testing correction using the Benjamini and Hochberg False Discovery Rate initially identified 269 transcripts which significantly (p<0.05) distinguished the two groups (Figure 1B). However, a modular interpretation of the gene expression revealed an unusual array of cellular expression and immune response in one of the children within the healthy control group. This child was excluded and the microarray statistical analysis was repeated without this subject (Figure 2). The completed analysis with 9 controls and 10 study patients identified 58 genes (24 over-expressed and 34 under-expressed) which significantly differentiated children with MRSA AHO from the healthy controls (Figure 3). The modular framework analysis of these children (Figure 4) demonstrated an up-regulation of innate immunity and downregulation of adaptive immunity. This was illustrated by significant over-expression of modules linked to neutrophil activity (M5.15), the coagulation cascade (M1.1), inflammatory response (M4.2), and erythrocyte development (M2.3). Concurrently, these children displayed an under-expression of modules associated with T-cells (M4.1, M6.15, and M6.19), cytotoxicity/NK cells and lymphocyte activation (M3.6 and M4.15), and B-cells (M4.10).

**Table 1:** Clinical Characteristics of Children Hospitalized with MRSA AHO During Study Time Period.

Subject Number	Age	Gender	Infectionsite	Bacteremia	Peak CRP (mg/dL)	Days to NormalCRP	Peak ESR (mm/hr)	Days to NormalESR	ICU admit	PICULOS(days)	TLOS (days)	Surgeries	Symptomonsetto admit(days)	Admit to culture positive (days)	Severity of Illness Score
1	13 yr	M	distal tibia	No	15.0	10	101	54	No	0	9	2	4	3	3
2	12 yr	M	distal femur	Yes	34.7	46	103	109	Yes	13	37	3	3	4	9
***3***	***12 mo***	***M***	***Ilium***	***Yes***	***34.1***	***30***	***138***	***59***	***No***	***0***	***23***	***2***	***4***	***6***	***8***
4	12 yr	F	distal fibula	No	26.8	17	88	45	No	0	9	1	6	3	5
5	8 yr	M	distal tibia	Yes	22.0	15	67	50	No	0	10	2	2	2	8
***6***	***14 mo***	***M***	***proximal humerus***	***Yes***	***20.5***	***29***	***108***	***60***	***No***	***0***	***33***	***4***	***5***	***3***	***7***
7	5 yr	M	distal tibia	No	2.6	20	99	68	No	0	9	1	7	2	0
8	3 yr	M	proximal and distal tibia	Yes	30.7	111	123	136	Yes	19	36	3	2	4	9
6	19 mo	M	distal femur	Yes	23.9	30	127	65	No	0	23	3	3	4	6
10	4 yr	M	distal tibia	Yes	16.1	20	71	52	No	0	10	1	2	3	3
11	12 yr	F	distal	Yes	55.6	61	100	115	No	0	21	2	5	2	8
12	28 mo	M	distal	Yes	25.2	37	91	65	No	0	17	1	4	2	7
				Mean values	25.6	35.5	101.3	73.2			19.8	2.1	3.9	3.2	6.0

Subjects excluded from statistical analysis in bold.

### Quantitative Gene Expression Analysis and Correlation with Severity of Illness

**Figure 5:**
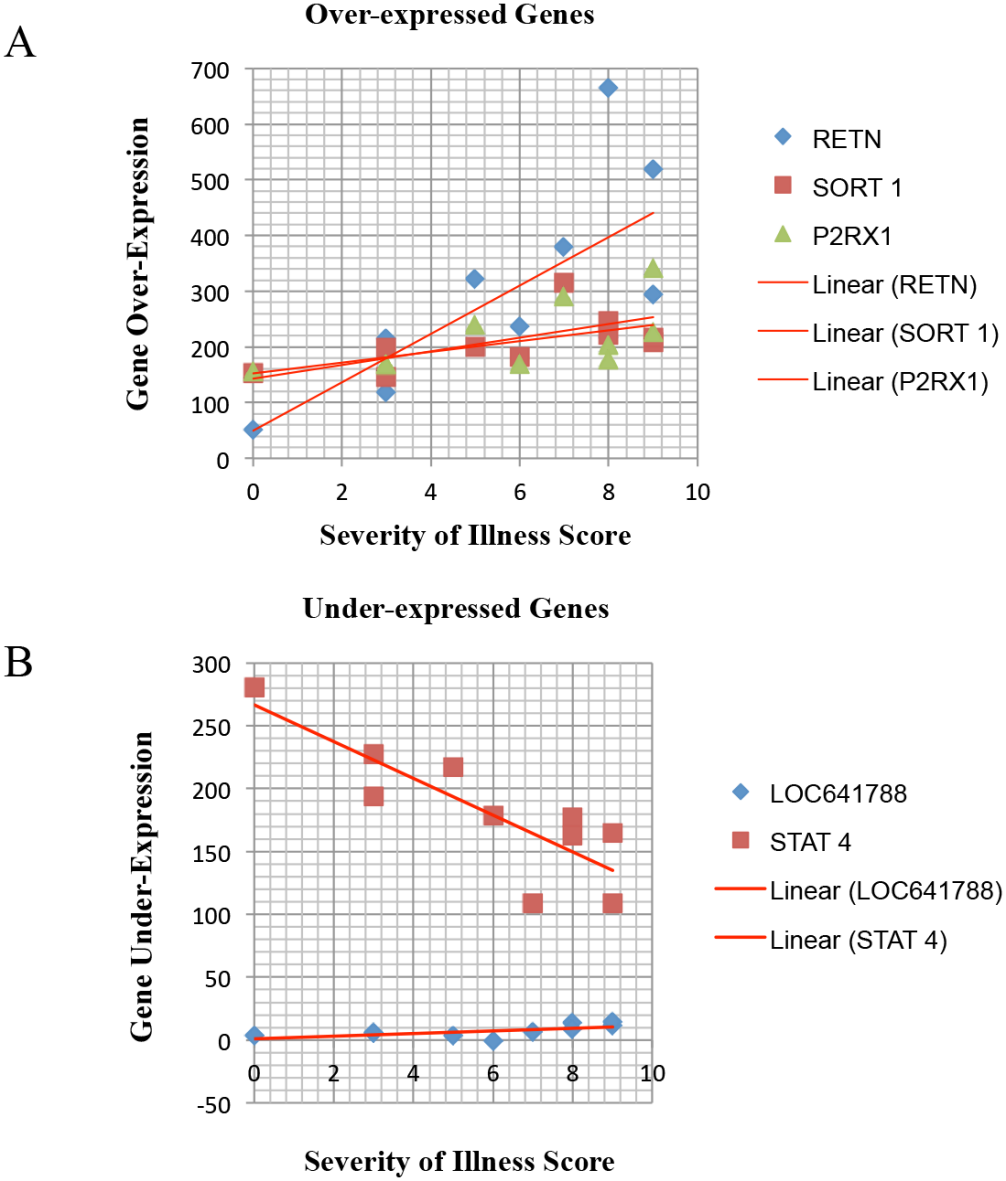
Correlation of Gene Over and Under-Expression and Severity of Illness Score (SIS). Statistical correlation (Pearson, Spearman, Pearson Log Gene and Pearson Log-Log) of severity of illness score and quantitative gene expression was significant for five genes. **A)** RETN (resistin gene), SORT 1 (sortilin 1 gene) and P2RX1 (purinergic receptor P2X, ligand-gated ion channel 1) showed over-expression. **B)** LOC641788 (similar to CDC26 subunit of anaphase promoting complex) and STAT 4 (signal transducer and activator of transcription 4) showed under-expression. Of those genes, RETN and STAT 4 had the strongest correlation coefficients (RETN: R2 = 0.8 and STAT 4: R2 = –0.83).

Severity of Illness Scores ranged from 0 to 9 with a mean of 6.1 and a median of 7 (Table 2). Table 3 lists the 58 genes associated with MRSA AHO and their quantitative expression relative to healthy controls. Statistical correlation of clinical disease severity and quantitative gene expression was done using Pearson, Spearman, Pearson Log Gene and Pearson Log-Log methods. Gene expression was found to significantly correlate with clinical Severity of Illness Score for five genes. Two genes, RETN and STAT 4 demonstrated significant correlation by all four statistical methods and had the strongest correlation coefficients (RETN: R^2^ = 0.8 by Pearson Log-Log and STAT 4: R^2^ = –0.83 by Pearson and Spearman). Three other genes were significantly correlated with severity of illness by three of the four statistical methods (P2RX1, SORT1, and LOC641788). Figure 5 illustrates the magnitude and direction of correlation of these five genes relative to severity of illness score.

**Table 2:** Severity of Illness Score (SIS) Calculation for Children Hospitalized with MRSA AHO During Study Time Period.

Subject	CRPInitial	CRP48 hrs	CRP96 hrs	RR Initial	RR % ofmid-range	Febrile dayson Abx	ICU Adm	DisseminatedDisease	Severity ofIllness Score
1	15	5.1	1.7	20	142%	0	0	0	3
2	34.7	24.2	30.7	22	92%	4	1	1	9
3	31.4	18.4	20.4	45	100%	3	0	1	8
4	26.4	26.8	7.1	20	83%	1	0	0	5
5	22	18.3	12.4	20	83%	3	0	1	8
6	18.2	20.5	16.1	40	125%	1	0	0	7
7	0.1	0.1	2.6	22	79%	0	0	0	0
8	17.7	21.7	27.1	28	88%	4	1	1	9
9	23.9	13.4	9.1	40	125%	1	0	0	6
10	16.1	9.3	3.2	28	100%	0	0	0	3
11	55.6	39.6	11.5	24	171%	3	0	0	8
12	22.6	12.1	10.8	32	100%	3	0	0	7
								Mean	6.08
**Severity of Illness Scoring Method with Mid-Range Normal Values of Respiratory Rate by Age.**									
**CRP initial**		**CRP** **48 hours**	**CRP** **96 hours**		**Respiratory rate**		**Respiratory rate mid-range** **normal values**		
<10 = 0		<5 = 0	<5 = 0		≥125% of mid-range norm = 1				
10–15 = 1		5–10 = 1	5–10 = 1		<125% = 0		Infant (birth-1 yr)		45
>15 = 2		>10 = 2	>10 = 2				Toddler (1–3 yr)		32
							Preschool (3–6 yr)		28
**Febrile days on Abx**			**ICU admission**		**Disseminated disease**		School-age (6–12 yr)		24
**within 96 hr of surgery**							Adolescent (12–18 yr)		14
<2 = 0			No = 0		No = 0				
≥2 = 1			Yes = 1		Yes = 1				

**Table 3:** Top 24 Over-expressed and 34 Under-expressed Genes in MRSA AHO.

Gene	Control Group	MRSA AHO Group	Fold Change
***RETN***	***42.4***	***237.51***	***7.63***
MAP1A	7.8	56.69	6.83
LCN2	434.9	2776.60	5.46
NDUFAF3	8.8	49.32	5.30
MYL9	30.8	139.25	5.30
CETP	18.3	89.58	4.97
PLOD2	14.6	63.07	4.45
SELP	24.0	87.03	3.52
PDGFC	13.0	40.43	3.34
MGLL	10.3	31.46	2.98
FAH	23.4	59.03	2.96
STOM	933.3	3204.18	2.85
C7ORF41	512.0	1478.31	2.65
CTSA	323.9	783.98	2.37
***P2RX1***	***103.5***	***199.40***	***2.18***
septin 5	3434.9	7817.88	2.13
VCL	1688.0	3471.43	2.03
C16ORF68	118.9	272.99	2.01
LOC100134188	15.8	32.83	1.83
G6PD	152.6	248.24	1.69
***SORT1***	***123.2***	***210.12***	***1.67***
NRD1	761.3	1186.23	1.57
CAPN1	344.0	532.86	1.55
ILK	1448.1	2262.23	1.51
FAM32A	531.7	358.97	–1.43
RAB7L1	199.0	139.64	–1.60
BBS2	119.3	62.17	–1.61
NR2C2AP	92.7	60.20	–1.63
BIRC3	113.8	72.10	–1.63
STK39	305.2	193.97	–1.64
***LOC100129237***	***220.5***	***134.92***	–***1.65***
GGPS1	305.9	184.71	–1.68
CD52	4585.5	2851.10	–1.70
SNHG6	1358.6	820.63	–1.71
ARRDC2	42.3	27.78	–1.76
CWF19L2	132.8	79.73	–1.76
LOC100128266	732.5	382.82	–1.84
TIGA1	585.2	340.31	–1.88
TOB1	280.5	158.49	–1.90
ZIK1	28.5	16.46	–1.93
***STAT4***	***329.4***	***176.98***	–***1.94***
RNF125	51.2	28.17	–1.96
LOC647346	86.6	45.71	–1.96
GIMAP1	400.7	213.03	–1.99
MAF	86.2	42.77	–2.26
C21ORF63	29.2	14.75	–2.28
HS.560343	113.7	59.16	–2.33
SIGIRR	109.2	45.85	–2.37
P2RY10	180.4	77.83	–2.42
TRMT11	55.4	23.72	–2.46
MRFAP1L1	26.2	6.44	–2.56
EBI2	446.0	189.88	–2.59
FAM84B	46.1	21.79	–2.71
LOC641788	19.8	4.15	–2.76
SLFN5	120.7	42.23	–2.80
HGD	94.2	36.64	–2.80
HS.558488	29.5	10.39	–2.88
PDZD4	32.3	11.30	–3.12

Genes associated to SIS in bold.

## Discussion

This study confirms a neutrophil pattern of gene expression of whole blood RNA among children with AHO caused by MRSA which has been previously described in children with invasive *S. aureus* infections. Furthermore it establishes a correlation of the expression of specific genes with clinical severity of illness in these children during the time of acute inflammation. In a similar manner to that of previous investigators, we have identified an up-regulation of gene expression pertaining to innate immunity concurrent with a down-regulation of gene expression pertaining to adaptive immunity. [13], [14] The insight gained from this series of children with a well-defined disease process is promising inasmuch as it suggests new directions to further explore the complex host-pathogen interaction in children with AHO.

Three previous studies have evaluated gene expression in children with *S. aureus* infections. [12]–[14] The first looked at 50 children with a variety of conditions including osteomyelitis –22, septic arthritis 6, pneumonia –6, lung abscess –1, endocarditis –1, abscess –9, cellulitis –1, disseminated –2 and bacteremia –2. [12] Both MRSA and MSSA infections were included in this study. Only 4 children with AHO from MRSA were included in the analysis. The study found 30 classifier genes, which differentiate children with *S. aureus* from those with *E. coli* infections. [12] However, they did not report their findings relative to normal controls. As such, our results are not directly comparable to theirs, not only due to the lack of a comparison to normal controls, but also due to the broad spectrum of conditions which they studied, including skin and soft tissue infections.

A second study evaluated children who also had a wide array of *S. aureus* infections in various patterns of involved tissues and systems Fifty-three children were included (osteomyelitis –37, septic arthritis –3, pneumonia –5, endocarditis –2, pyomyositis –1, abscess –2, lymphadenitis –1, and bacteremia –2) with both MRSA (31) and MSSA (22) isolates represented. [13] There were 8 children with AHO from MRSA. [13] The investigators found that *S. aureus* infections were associated with over-expression of innate immunity and under-expression of genes related to adaptive immunity, which is consistent with the findings of our study. [13] We found three over-expressed genes in common with the study of Ardura et al.: LCN2, MYL9, and RETN. [13] It is important to note that both of the aforementioned used PBMC transcription, rather than assessing whole blood RNA [12], [13].

One other study reported whole blood RNA sequencing for children with *S. aureus* infections. [14] The study included a mixture of infection types (superficial, invasive, and disseminated) and systems involved (osteoarticular and pneumonia). It also contained a combination of MRSA and MSSA isolates. The results echoed the finding of the PBMC microarray studies with regard to innate immunity up-regulation including the myeloid lineage and adaptive immunity down-regulation. The investigators did correlate gene expression patterns with the heterogenous clinical presentations of the infected children according to a variety of laboratory parameters. [14] However, their use and interpretation of these parameters differs substantially from ours. Of note, the draw day for the Tempus tubes in their study ranged widely from day 1–35. For these several reasons, our results are not directly comparable.

To the best of our ability, we controlled the population being studied by exclusively including children with osteomyelitis due to a solitary organism (MRSA). Each of the children presented to the hospital within 3 days of the onset of symptoms. The diagnostic MRI which led to the decision for surgery was accomplished by protocol within 24 hours of triage. The culture material sent was positive for MRSA within 48 hours of specimen delivery to the microbiology laboratory. Finally, the whole blood samples were drawn on the day that culture results were confirmed as positive (within 4 days of hospitalization). Given the proximity of these events for each of the children within this study, we consider that this series represents the most homogenous cohort studied among them.

A limitation of our study is the small sample size; additionally 2 patients with MRSA AHO were removed from the analysis due to unavailable matched control samples. Patients 3 and 6 were the two study subjects excluded. They were the youngest in the group with corresponding ages 12 and 14 months. Those two study subjects had high severity of illness score (8 and 7) and similar clinical characteristics (Table 1). It is difficult to predict the impact of their exclusion from subsequent analysis. Nonetheless, it is important to consider that study subjects number 9 and 12 had similar ages and severity of illness scores, thus representing this age group in the study sample.

When normalization was done using healthy controls expression values, there was a healthy control that was classified far away from the rest of healthy controls. This control had unique signature which was compatible with a viral infection. Due to this, the control subject was excluded from subsequent microarray statistical analysis.

There is an opportunity afforded, by the presentation of our findings, to conduct focused assays on a larger cohort of children at lower cost by evaluating the expression of a smaller gene list. An important clinical question is whether or not the gene over- or under- expression follow a temporal pattern of normalization consistent with the clinical resolution of disease. This type of study would be challenging in a large series of children if whole blood gene expression was to be accomplished at multiple time intervals.

There is limited information reported about the genes which we found to be significantly correlated with clinical severity of illness. Resistin (RETN) is a systemic immune-derived pro-inflammatory cytokine targeting leukocytes. [16], [17] In humans, resistin is found in a variety of different tissues, although the highest level of expression is in the bone marrow. [18], [19] Relevant to our research, resistin has been shown to be an important regulator of the inflammatory cytokine cascade, exerting its pro-inflammatory effects through the NF-kB signaling pathway. [20]–[22] Human neutrophils are an important source of resistin and its release and production are upregulated by pro-inflammatory signals acting on mononuclear leukocytes as well as certain bacterial stimuli. [23]–[24] Human leukocyte resistin expression depends on the myeloid specific nuclear transcription factor CCAAT enhancer binding protein epsilon (CEBPE). [17] Resistin has been shown to accumulate at sites of inflammation and its levels correlate with other acute phase reactants. [25]–[27] Resistin also appears to be a chemo-attractant for CD4+ cells and plays a role in inhibiting chemotaxis and decreased oxidative burst in neutrophils. [18], [28], [29] Additionally, resistin can induce secretion of inflammatory cytokines such as IL 6, IL8, and TNF from PMBCs and it has been linked to both acute and chronic inflammatory states as rheumatoid arthritis and endotoxemia. [22], [26], [30], [31] Systemic levels of resistin have previously been correlated to severity of disease in patients with severe sepsis [31].

P2RX1 belongs to the family of purinoceptors for ATP. This receptor functions as a ligand-gated ion channel with relatively high calcium permeability. [32], [33] Human neutrophils have been found to express functional P2RX1 channels and activation of these channels by ATP binding has been shown to trigger Rho dependent signal amplification, which is involved in promoting neutrophil migration toward a chemo-attractant stimulus. P2RX1 channels appear to have a protective role in endotoxemia by negatively regulating undesired neutrophil activation. This limits the oxidative response, and minimizes coagulation and end organ damage associated with septic shock while allowing more efficient chemotaxis towards the sites of inflammation and infection [34], [35].

SORT1 encodes the protein sortilin, a multi-ligand type-1 receptor produced in the liver. [36] Sortilin has been shown to negatively regulate TGF-β signaling. [36]–[38] The TGF-β family plays an important role in the body’s immune response by increasing the proliferation, differentiation, migration and survival of immune cells, especially T lymphocytes through its regulation of transcription of target genes via ligand mediated receptor binding (TGF-β-3) [39]–[42].

LOC641788 encodes a protein similar to the CDC26 subunit of the anaphase promoting complex (APC), an essential cell-cycle regulator. [43] It regulates by promoting ubiquitin-mediated proteolysis of key cell cycle regulatory proteins. Finally, STAT 4 is a signal transducer and activator of transcription. [44]–[46] STAT4 activation is induced by IL-12 receptor engagement and in turn promotes TH1 responses that produce IFNγ. IFNγ then send signals via STAT1 to increase anti-tumor responses through the activation of natural killer cells, macrophages and CD8+ T cell-mediated cytolytic activity. STAT4 also has a direct regulatory effect on the production of the perforin protein, involved in NK cell and cytotoxic T cell mediated cytolysis, via the perforin gene. Without the presence of STAT4, this protein is not produced by immune cells. STAT4 was found to be downregulated in our cohort of patients with AHO. This could potentially explain our findings of under-expression of genes related to adaptive immunity.

Beyond the specific function of these expressed genes is the potential for pattern recognition during the early course of disease. A child who is evolving clinically toward severe illness could possibly be differentiated from another who is not by identifying the pattern of over- and under- expression of a small subset of genes, before the actual clinical manifestations are identified. In addition, this early detection would provide physicians a framework from which to base their therapeutic decisions, including starting out with a more aggressive treatment course in children who are more likely to develop severe disease, hopefully decreasing the time and emotional stress involved in a prolonged hospital stay. Novel diagnostic tools, using rapid polymerase chain reaction (PCR) to assess the quantitative over- or under-expression of these genes in isolation or combination would potentially have prognostic significance and direct resources of care and attention toward children who need them the most.

An important question which this study raises is whether or not the gene expression of children with AHO follows a pattern parallel to clinical activity of the disease over time and treatment course. It would be beneficial to determine if there a normalization timeframe reflective of resolution of the infection or if residual or chronic foci of infection were present. Our current tools for monitoring disease resolution in AHO, such as erythrocyte sedimentation rate and the clinical impression of a sufficient duration of antibiotic treatment, are rudimentary and arbitrary. There is increasing evidence that disease activity in children with systemic lupus erythematosus may be monitored with microarray analysis and influences clinical intervention. In order for this to be accomplished for children with AHO, a much larger series would need to be studied over a longer period of time, with multiple blood samples taken at various time intervals. Unfortunately, the current cost of microarray processing makes this type of study challenging without a narrower list of genes to study using more focused techniques, such as PCR.
